# 

**Published:** 1845-04

**Authors:** 


### THE ILLINOIS MEDICAL AND SURGICAL JOURNAL

### PUBLISHERS' NOTICE.

The publishers of the Illinois Medical and Surgical Journal, are happy to announce to their subscribers, that they will continue its publication as before, issuing the first number of the second volume in April. They take this opportunity to return their thanks for the increasing patronage which the Journal has received at the hands of the medical public and others, and confidently trust in the merits of the work, to sustain itself and extend its usefulness.

The Journal will be continued upon the same terms as before, will be issued monthly, each monthly number containing 16 pages octavo, with an index and title page in the last number.

It will still be conducted, in the editorial department, by James V. Z. Blaney, M. D., Professor of Chemistry and Pharmacy, in the Rush Medical College.

Chicago, March, 1845.

### TO SUBSCRBERS.

THE PUBLISHERS feel themselves obliged to call upon the subscribers to the Journal *to remit the amount of their subscriptions.* Though the subscriptions are required *in advance*, there are a large number of names upon the list not yet marked paid. The price of the Journal is so low that they cannot conceive that this arises from other cause than neglect. Postmasters have the power and are always willing to frank remittances. The publishers hope that they will not be obliged again to issue this notice, or be driven to the disagreeable alternative of stopping the issue of the Journal to those who have not paid their subscriptions.

ELLIS & FERGUS, PUBLISHERS.

